# Characteristics of the Fiber Laser Sensor System Based on Etched-Bragg Grating Sensing Probe for Determination of the Low Nitrate Concentration in Water

**DOI:** 10.3390/s17010007

**Published:** 2016-12-22

**Authors:** Thanh Binh Pham, Huy Bui, Huu Thang Le, Van Hoi Pham

**Affiliations:** 1 Institute of Materials Science, Vietnam Academy of Science and Technology, 18 Hoang Quoc Viet Rd, Cau giay District, Hanoi 100000, Vietnam; buihuy@ims.vast.ac.vn (H.B.); hoipv@ims.vast.ac.vn (V.H.P.); 2 Small and Medium Enterprise Development and Support Center 1, Directorate for Standards, Metrology and Quality, 8 Hoang Quoc Viet Rd, Cau giay District, Hanoi 100000, Vietnam; lhthang2001@gmail.com

**Keywords:** nitrate, etched-Fiber Bragg Grating, fiber laser, optical sensor

## Abstract

The necessity of environmental protection has stimulated the development of many kinds of methods allowing the determination of different pollutants in the natural environment, including methods for determining nitrate in source water. In this paper, the characteristics of an etched fiber Bragg grating (e-FBG) sensing probe—which integrated in fiber laser structure—are studied by numerical simulation and experiment. The proposed sensor is demonstrated for determination of the low nitrate concentration in a water environment. Experimental results show that this sensor could determine nitrate in water samples at a low concentration range of 0–80 ppm with good repeatability, rapid response, and average sensitivity of 3.5 × 10^−3^ nm/ppm with the detection limit of 3 ppm. The e-FBG sensing probe integrated in fiber laser demonstrates many advantages, such as a high resolution for wavelength shift identification, high optical signal-to-noise ratio (OSNR of 40 dB), narrow bandwidth of 0.02 nm that enhanced accuracy and precision of wavelength peak measurement, and capability for optical remote sensing. The obtained results suggested that the proposed e-FBG sensor has a large potential for the determination of low nitrate concentrations in water in outdoor field work.

## 1. Introduction

Nitrate (${\mathrm{NO}}_{3}^{-}$) is considered to be one of the important substances to measure in water, because of its potential environmental and human health implications. The main anthropogenic sources of nitrates in the environment are municipal and industrial waste, artificial fertilizers, septic systems, animal feedlots, and food processing waste such as food preservatives, especially to cure meats. Nitrates can cause eutrophication of surface waters. Nitrates are not directly toxic to human health, but their possible reduction to nitrites and a next reaction of nitrites with secondary or tertiary amines present in the body can result in the formation of carcinogenic N-nitrosamines. Moreover, the nitrite oxidizes iron in the hemoglobin of the red blood cells to form methemoglobin, which lacks the oxygen-carrying ability of hemoglobin. This creates a condition known as methmoglobinemia, wherein blood iron in hemoglobin (Fe^+2^) is reduced to its oxidized form Fe^+3^. Different methods, including ultraviolet-visible spectroscopy (UV-VIS), electrophoresis, electrochemical detection, chromatography, mass spectroscopy, and potentiometry coupled with sequence injection analysis are adopted in finding the concentration of nitrate [1,2,3,4,5]. These methods are expensive and/or inconvenient for field work.

Optical fiber sensors offer very attractive solutions over conventional technologies due to some unique characteristics such as multiplexing capabilities, high sensitivity, fast response, and immunity to electromagnetic interference. The small physical size of optical fiber allows the development of very small and flexible fiber sensors, and enables the remote in-situ sensing of species in difficult or hazardous environments [6,7]. Optical fiber sensors based on colorimetric technique [8,9] and evanescent wave absorption [10] for in-situ nitrate detection in water have been proposed. These methods can detect the nitrate concentration in the range from ppb to ppm, but the measurement response time is some tens of minutes.

Fiber Bragg gratings (FBGs) have been demonstrated as optical sensors for various applications [11,12,13], especially for chemical and biochemical sensing [14,15,16,17]. In various chemical and biochemical applications, refractive index sensing is important, since several substances can be detected by the measurement of the refractive indices. The FBG sensing operation principle relies on the dependence of the Bragg resonance wavelength on the grating pitch and effective refractive index. Normal FBGs are intrinsically insensitive to the ambient refractive index. However, if the fiber cladding diameter is reduced along the grating region, the effective refractive index is significantly affected by the external refractive index. Among different kinds of FBG, the Tilt-FBG and the Long Period Fiber Grating (LPFG) have shown a large potential for chemical and bio-sensing applications with high sensitivity and low cost. However, their multiple resonance peaks limit their multiplexing capabilities. Moreover, the measurement accuracy of LPFG is limited due to its broad line-width at full-width at half maximum (FWHM) [18].

The aim of our study is investigation of characteristics of etched-fiber Bragg grating (e-FBG) sensing probe integrated in fiber laser structure as lasing wavelength selected element for determination of low nitrate concentrations in water. The e-FBG sensing probe is designed and fabricated by wet chemical etch-erosion and put into a fiber cavity laser using Er^+3^-doped silica fiber. In the interaction between the evanescent wave of the fundamental core mode and the surrounding medium, a small variation of the refractive index of the medium rounding the e-FBG will induce a significant change in the Bragg wavelength according to the Bragg condition, and the response time of measurement is less than a milli-second. The e-FBG sensing probe can be used to detect the nitrate in water samples at a low concentration range of 0–80 ppm. The line-width spectrum of lasing emission from a fiber laser is much narrower than that of reflected FBG spectra, thus enhancing the detection accuracy and capability for remote sensing.

## 2. Experiment

**Figure 1:**
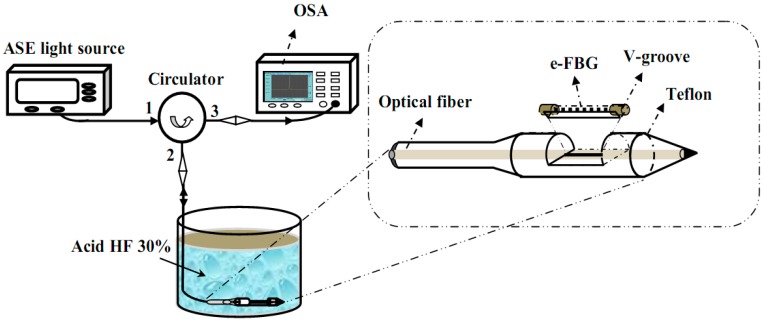
Experimental setup and mount design for making etched-fiber Bragg grating (e-FBG). ASE: amplified spontaneous emission; OSA: optical spectrum analyzer.

The FBGs used in our experiments were fabricated with the standard single-mode photosensitive fiber (Model: PS 1250/1500, Fiber-core, Southampton, UK) by the Talbot interferometric technique with exposure to the KrF Excimer Laser source of 248 nm wavelength (ASX-750 Excimer Laser, MPB Technology Inc., Montreal, QC, Canada). The Bragg resonant wavelength was 1550 nm with 12 mm long FBG, and the reflection line width at FWHM was 0.2 nm [12]. The e-FBG was fabricated by wet chemical etching the FBG region in hydrofluoric acid (HF) solution to increase the interaction of the propagating optical field in the fiber core with the surrounding medium. The etching technique was performed in two steps: the first step used a 30% HF solution to speed up the etching fiber cladding layer process. After an etching process for 75 min, the fiber diameter was below 15 µm. The etching solution was then replaced with a 15% HF solution for the second step. The purpose of the second step is to slow down the etching process and smoothing the etched fiber surface (for 20 min). A schematic diagram of the experimental setup for fiber-mount design and for measuring the reflected Bragg wavelength shift of e-FBG in the etching process is shown in Figure 1. A broadband light source from amplified spontaneous emission (ASE) of Erbium-doped fiber amplifier, an optical circulator, and an optical spectrum analyzer (OSA: Advantest Q8384 with a resolution of 0.01 nm) are used for monitoring the wavelength shift. The input ASE signal passes through a circulator before being reflected by the FBG and directed to an OSA. There is a need to have a protective mount for the fragile e-FBG with small diameter of micrometers, so the design of the FBG-mount is also shown in Figure 1. Before the corrosion process, the FBG is mounted and fixed at two ends with epoxy on Teflon V-groove mount, which is non-reactant to HF solution and decreased mechanical vibration for e-FBG.

The fiber laser using e-FBG as a reflector (which operates as an optical sensor) was proposed for the determination of low nitrate concentration in water. The optical gain medium was an erbium-doped silica fiber (Model: EDF-HCO-4000, Core-active, Quebec, QC, Canada) with length of 3 m, and the optical pump was a 980 nm-laser diode with output optical power up to 170 mW in single-mode emission (SDLO-2564-170). The pumped light was through a 980/1550 nm wavelength division multiplexer (WDM) to Er-doped silica fiber for excitation of the erbium ions. The other end of the erbium doped fiber was connected to an e-FBG sensing element as a mirror of fiber laser system. A fiber-optic circulator was used to couple the light into the cavity and through an optical coupler 10/90 in order to extract 10% of the light from the cavity to the acquisition system, and 90% of the light comes back to the cavity. This fiber laser configuration will give narrow line-width of lasing emission and high optical signal-to-noise ratio. The spectral characteristics of the lasing emission were analyzed by the OSA. The e-FBG sensing probe was immersed in solvent, and the reflection spectrum from the e-FBG sensing element was changed by different solutions of nitrate concentration varying from 0 to 80 ppm. The potassium nitrate stock solution was prepared by dissolving 0.4075 g of anhydrous KNO_3_ (Merck) in purified water (250 mL) to obtain a sample of 1000 ppm nitrate in water. One hundred milliliters of this solution was diluted to one litre of water to produce the stock 100 ppm solution. By this dilution method, we obtained nitrate solutions with low concentration from 0 to 80 ppm for use in our experiment. All the measurements were done at constant temperature of 25 °C.

## 3. Results and Discussion

**Figure 2:**
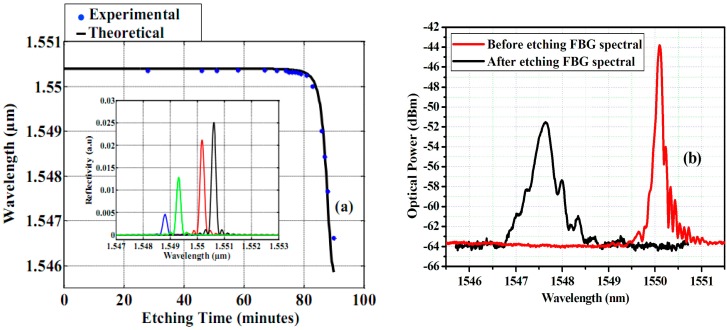
(**a**) Wavelength shift of FBG versus the etching time; and (**b**) reflected spectra of FBG before and after etching process.

During the etching process, the shift of Bragg wavelength was monitored at regular intervals by recording the reflected spectrum peak from the FBG in different moments (shown in Figure 2a). As time progressed, the Bragg wavelength shifted to the shorter wavelength range (blue shift) due to the reduction of the cladding diameter, since the fundamental mode is less confined in the fiber core region, leading to a higher evanescent field, and thus to a more efficient interaction with the surrounding medium. For the mechanical strength and the durability of e-FBG for practical use, we have limited the etched fiber diameter to 6–8 µm.

In common FBG, the effective refractive index of the fundamental mode does not practically depend on the refractive index of the medium surrounding the fiber. However, if the cladding diameter is reduced, this effective refractive index shows a nonlinear dependence on the external refractive index and leads to a shift in the reflected wavelength. The effective refractive index in an e-FBG is evaluated by numerically resolving the dispersion equation of a double-clad fiber model. According to the theory of the fiber Bragg grating, the Bragg wavelength *λ_B_* is as follows:

(1) $$\lambda_{B}=n_{eff}\Lambda$$

where *n_eff_* and Λ are the effective refractive index and periodic spacing of FBG, respectively. According to the optical fiber Coupled-Mode Theory, the relationships between the effective refractive index of the e-FBG, fiber diameter, and the normalized frequency *V_ext_* of the etched single-mode fiber are follows [16]:

(2) $$n_{eff}^{2}=n_{co}^{2}-{\left(\frac{U}{V_{ext}}\right)}^{2}\left(n_{co}^{2}-n_{cl}^{2}\right)\;U=a\sqrt{k_{0}^{2}n_{co}^{2}-\beta^{2}},\;V_{ext}=\frac{\pi d}{\lambda }\sqrt{n_{co}^{2}-n_{ext}^{2}}$$

where *a* and *d* are the fiber core radius and the e-FBG diameter, respectively; *n_co_*, *n_cl_*, and *n_ext_* are the refractive indexes of the fiber core, cladding, and external medium, respectively; *k*_0_ = 2*π/λ* is vacuum wave number; and *β* is a propagation constant. The reflection wavelength shift of e-FBG is only related to the effective refractive index. The simultaneous differential equation from Equations (1) and (2) is as follows:

(3) $$\frac{\Delta \lambda_{B}}{\lambda_{B}}=\frac{\Delta n_{eff}}{n_{eff}}=\frac{U^{2}\left(n_{co}^{2}-n_{cl}^{2}\right)}{2V_{ext}^{3}\left[n_{co}^{2}-{\left(\frac{U}{V_{ext}}\right)}^{2}\left(n_{co}^{2}-n_{ext}^{2}\right)\right]}\;$$

**Figure 3:**
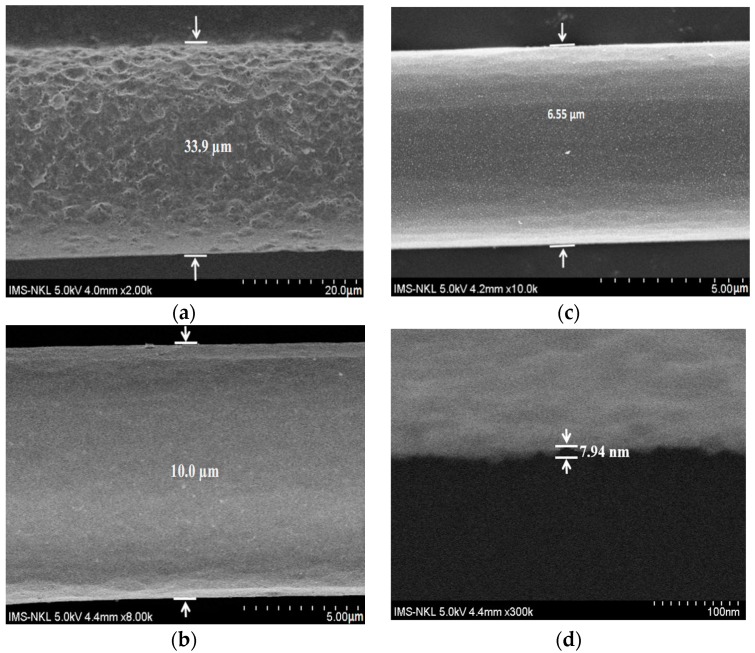
SEM images of e-FBGs with diameters of: (**a**) 33.9 µm; (**b**) 10 µm; (**c**) 6.55 µm; and (**d**) of e-FBG surface with roughness of 7.94 nm.

In our calculation, the fiber parameters were chosen as: *n_co_* = 1.45, *n_cl_* = 1.4464, *d* = 4.5–125 µm, Λ = 0.53472 µm. The response simulation spectra of e-FBG (in the inset of Figure 2a), numerical predictions, and experimental values of the reflection wavelength shift of e-FBG during the etching process are shown in Figure 2a. It is observed that the calculated result fits very well to the experimental one. The reflected spectra of the original FBG and e-FBG obtained experimentally are shown in Figure 2b. When the diameter of fiber etched was 6.55 µm, the wavelength shift was 2.56 nm—this value is very close to the one provided by the numerical analysis by Equation (3) in the case of quasi-full etching. The experimentally-obtained spectral line-width increased from 0.2 nm to 0.7 nm between before and after etching grating. This ensures that the thickness of the FBG has reached core level, as shown in Scanning Electron Microscopy (SEM) images for produced e-FBGs with diameters of 33.9, 10, and 6.55 µm. Figure 3a shows an SEM image of e-FBG as it performed corrosion of the first step. It is observed that the e-FBG surface has large roughness, enabling it to induce adjacent modes and decrease intensity of the FBG reflection spectrum due to light scattering. Figure 3b,c shows SEM images of the final two produced e-FBGs after corrosion by the second step to be completed with a smoothed surface of the e-FBG. Figure 3d shows an SEM image of the e-FBG surface with roughness of about 7.94 nm corresponding to *λ*/194 (*λ*: 1550 nm wavelength of light transmitted in sensor system). This fine smoothness of the fiber surface will decrease evanescent wave scattering at the fiber surface, and we can obtain the high intensity of lasing emission.

**Figure 4:**
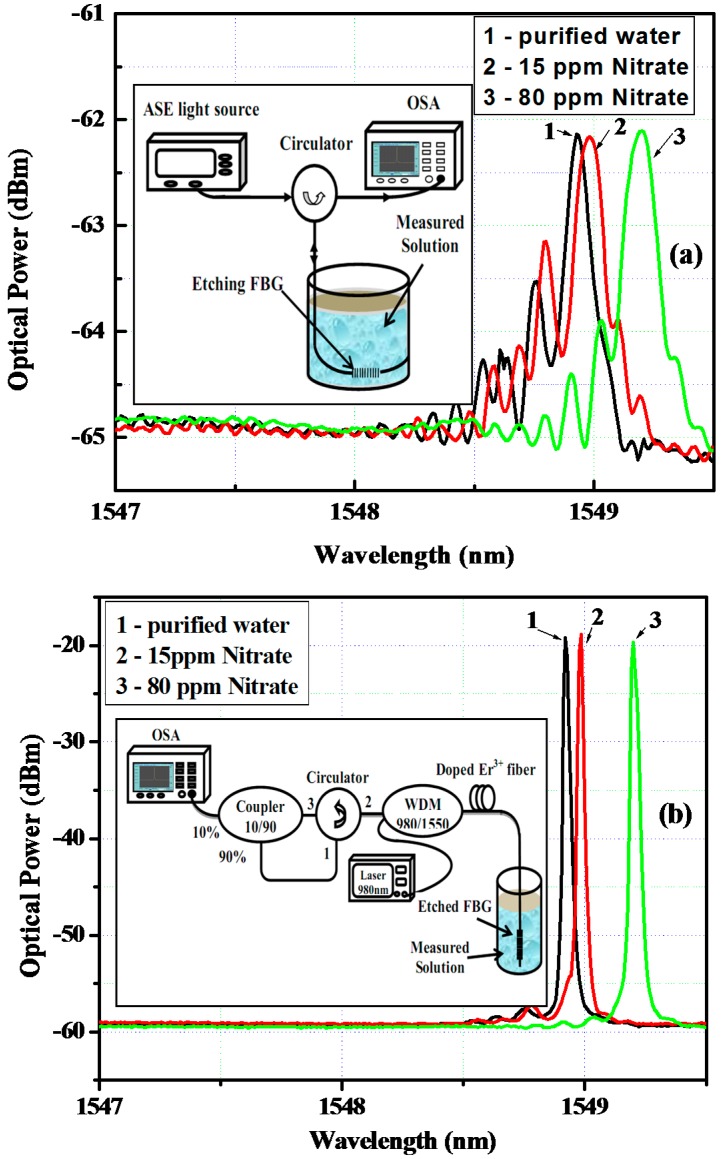
The spectral responses of (**a**) e-FBG from reflected configuration; and (**b**) of e-FBG integrated fiber laser configuration. The −3 dB-bandwidths of spectra decreased from 0.55 nm to 0.02 nm, and optical signal-to-noise ratio (OSNR) increased from 3 dB to 40 dB.

The spectra of reflection light from e-FBG sensing element and of lasing emission from fiber laser used the same e-FBG element as reflector experimentally obtained for different solutions of nitrate concentration in water measured on the OSA are shown in Figure 4a,b, respectively. The lasing emission from e-FBG integrated erbium-doped fiber laser has an optical signal-to-noise ratio (OSNR) higher than 40 dB and spectral line-width of 0.02 nm at −3 dB, whereas OSNR and spectral line-width of reflection light from e-FBG sensing element are about 3 dB and 0.55 nm, respectively. These narrow line-width and high OSNR characteristics of fiber laser emission will give high accuracy and high sensitivity for wavelength peak measurement method. In addition, the high optical intensity from the laser can be transmitted through the fiber for a long distance, which is required for remote sensing systems.

From the characteristic spectral response of the e-FBG sensing element, the signal-to-noise ratio (SNR) of the e-FBG sensor can be assumed to be inversely proportional to e-FBG spectral line-width, defined as [19]:

(4) $$SNR\left(n_{s}\right)={\left[\frac{\Delta \lambda_{res}}{\Delta \lambda_{SW}}\right]}_{n_{s}}$$

where ∆*λ_res_* is the resonance wavelength shift induced by the e-FBG sensing element, and ∆*λ_SW_* can be calculated as the full width at half maximum (FWHM) of the spectral response of the e-FBG sensing element. The *SNR* of the e-FBG sensor depends on how accurately and precisely the e-FBG sensor can detect the resonant wavelength shift of the sensing element. Therefore, the characteristic spectral response of the fiber laser-based sensor system has shown much narrower spectral line-width in comparison with e-FBG-based sensors, such that the fiber laser using e-FBG sensing element sensor system has strongly enhanced detection accuracy when the wavelength peak measurement is used.

**Figure 5:**
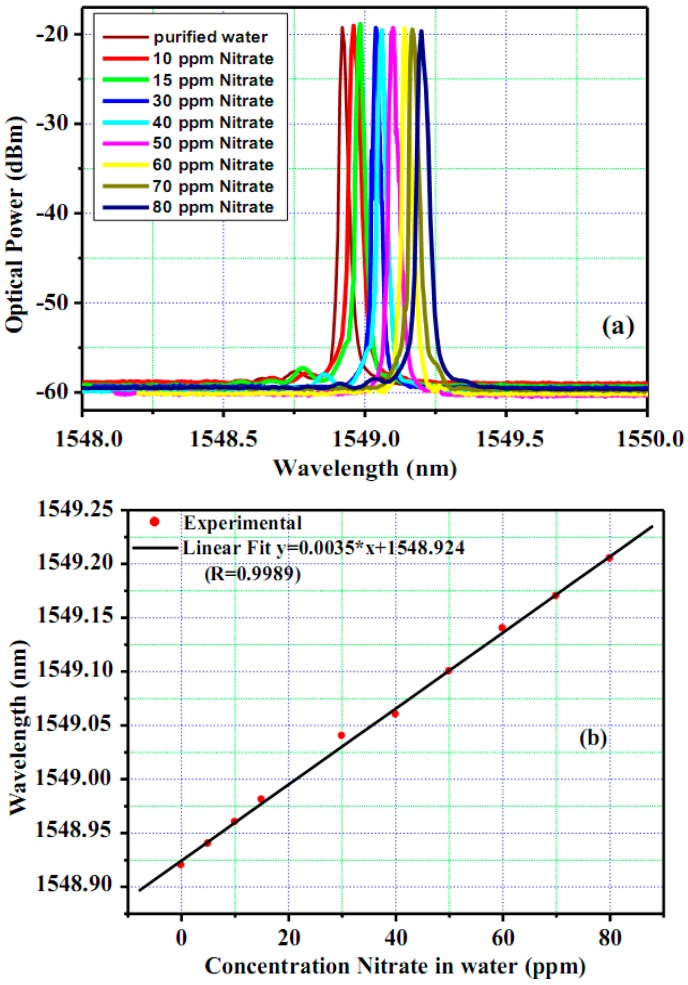
(**a**) Spectral response for different concentrations of nitrate solutions from fiber laser sensor; and (**b**) Bragg wavelength shift as a linear function of nitrate concentrations in water.

In order to demonstrate in detail the determination of nitrate concentration in water using an e-FBG integrated fiber laser sensor, the proposed sensor has been performed for the detection of nitrate in the concentration range of 0–80 ppm with step changes of 5–10 ppm. The designed e-FBG sensing probe was cleaned off the residual test sample solution by the de-ionized water before replacing the different test sample to avoid contamination and to ensure the accuracy of the measurement results. All the measurements were done at constant temperature 25 °C, and a standard deviation of the wavelength shift was obtained from the average value of five experimental data runs. The experimental results are depicted in Figure 5. 

The lasing spectral response of the e-FBG sensing element corresponding to different nitrate concentrations in water is shown in Figure 5a. The spacing between the peaks of the lasing spectra can easily distinguish with OSA resolution of 0.01 nm. It is observed that the increase of nitrate concentration of the water environment caused the lasing wavelength to shift to longer wavelength range (red shift). From Figure 5b, the wavelength shift of the lasing emission was 0.3 nm when the nitrate concentration in water changed from 0 ppm to 80 ppm. The calculated slope of linearly fitted data can be used as the effective sensitivity of the sensor; it can be deduced that the sensitivity of this sensor is achieved to 3.5 × 10^−3^ nm/ppm. Therefore, assuming that the detectable spectral resolution of OSA is 0.01 nm, the optical sensor can measure nitrate concentrations in water with a detection limit of 3 ppm.

Table 1 shows the results of detection limit and response time of nitrate measurement using different sensing techniques, such as electrochemical sensors with different electrodes, colorimetric, evanescent wave absorption fiber sensors, and e-FBG integrated to fiber laser.

It is remarkable that the proposed e-FBG sensor is a typical physical sensor without functionalized materials on the surface, which has the fast response time of in-situ refractive index measurement of aqueous environment, and it is suitable for reversible use by easily cleaning the glass surface. In addition, the silica glass-based sensor has good repeatability and reproducibility in the aqueous medium from its non-corrosion and stable properties in this environment. The limit of detection of current sensor (3 ppm) is far below the maximum nitrate level allowed in drinking water by the United States Environmental Protection Agency (EPA) [23], giving it large potential for application in monitoring drinking water.

## 4. Conclusions

We successfully designed and prepared the fiber laser using e-FBG sensing element as reflector and used this device to detect nitrate concentration in water. This sensor provides a new approach for real-time in-situ measurement with high accuracy. Moreover, this proposed sensor system shows many advantages including a high resolution for wavelength shift identification, high OSNR and narrow band-width that enhances accuracy and precision of wavelength peak measurement and improves capability for remote sensing application. To confirm the feasibility of the determination of nitrate concentration in water, experimental results demonstrate the usefulness of the e-FBG sensor in measuring nitrate compounds in water with good sensitivity in the low concentration range of 0–80 ppm with detection limit of 3 ppm. This established that the proposed sensor can be used for monitoring the water quality in field work. This sensor has a large potential for applications in agriculture, industrial fluids, and the food industry.

## Figures and Tables

**Table 1:** Comparison of nitrate-in-water detection limits and response times using different sensors.

Type of Sensors	Limit of Detection (ppm)	Measured Response Time	References
Electrochemical sensor	1.35	Tens of minutes	Liang et al. [20]
Graphene oxide sensor	0.05	30 min	Ren et al. [21]
Disposable Electrochemical sensor	8.6	Not reported	Bui et al. [1]
Evanescent wave absorption Fiber sensor	0.06	Not reported	Kumar et al. [10]
Colorimetric sensor	4.0	30 min	Kunduru et al. [9]
Lopine sensitive layer Fiber sensor	1.0	40 milliseconds	Camas-Anzueto et al. [22]
e-FBG in fiber laser sensor	3.0	Milliseconds	This work
